# Magnesium-Based Bioactive Composites Processed at Room Temperature

**DOI:** 10.3390/ma12162609

**Published:** 2019-08-16

**Authors:** Moara M. Castro, Debora R. Lopes, Renata B. Soares, Diogo M. M. dos Santos, Eduardo H. M. Nunes, Vanessa F. C. Lins, Pedro Henrique R. Pereira, Augusta Isaac, Terence G. Langdon, Roberto B. Figueiredo

**Affiliations:** 1 Department of Metallurgical and Materials Engineering, Universidade Federal de Minas Gerais, Belo Horizonte 31270-901, Brazil; 2 Department of Chemical Engineering, Universidade Federal de Minas Gerais, Belo Horizonte 31270-901, Brazil; 3 Materials Research Group, Department of Mechanical Engineering, University of Southampton, Southampton SO17 1BJ, UK

**Keywords:** bioactive glass, biodegradable material, composites, high-pressure torsion, hydroxyapatite, magnesium

## Abstract

Hydroxyapatite and bioactive glass particles were added to pure magnesium and an AZ91 magnesium alloy and then consolidated into disc-shaped samples at room temperature using high-pressure torsion (HPT). The bioactive particles appeared well-dispersed in the metal matrix after multiple turns of HPT. Full consolidation was attained using pure magnesium, but the center of the AZ91 disc failed to fully consolidate even after 50 turns. The magnesium-hydroxyapatite composite displayed an ultimate tensile strength above 150 MPa, high cell viability, and a decreasing rate of corrosion during immersion in Hank’s solution. The composites produced with bioactive glass particles exhibited the formation of calcium phosphate after 2 h of immersion in Hank’s solution and there was rapid corrosion in these materials.

## 1. Introduction

Biodegradable metals provide an opportunity for fabricating temporary implants with high initial load-bearing capacities that degrade as the surrounding tissue regenerates. In principle, the degradation byproduct can be absorbed and excessive amounts expelled by the human body without the need for secondary surgery for removal. A recent review describes past and current clinical trials in this field [1] where orthopedic and cardiovascular applications are the main targets. Iron, magnesium, and zinc display good biocompatibility and gradually degrade in physiological media. Iron exhibits the highest mechanical strength, but the corrosion rate is too low, whereas zinc exhibits a fast corrosion rate but poor mechanical properties. Magnesium has attracted significant attention for these applications despite the relatively low strength of the pure metal and the fast corrosion of its alloys. The maximum daily dosage intake of magnesium is high [2] and the elastic modulus is low where these are considered advantages for this material.

In addition to biocompatibility, a bone graft should exhibit mechanical integrity with the bone tissue. This means that the mechanical strength and fracture toughness should be high and the Young’s modulus must be similar to that exhibited by bone [3]. Together with its biological characteristics of high biocompatibility and a non-toxic risk during biodegradation, the density (1.74 g/cm^3^) and tensile strength (~135–285 MPa) of magnesium are similar to those of bone (1.8–2.0 g/cm^3^ and ~35–283 MPa, respectively) [3]. In addition, magnesium displays a higher fracture toughness of ~15–40 MPa/m^2^ compared with ~3–6 MPa/m^2^ for bone. The similarity in density and elastic modulus to bone makes magnesium a promising candidate material for applications such as bone graft because these properties will lead to lower stresses at the implant-bone interfaces [4].

In order to improve the performance of magnesium-based materials, research has focused primarily on improving the mechanical strength and corrosion resistance. Alloying is an effective way to increase the yield strength, but usually this will compromise the corrosion. Alternatively, grain refinement provides an opportunity for improving the strength without changing the composition. Severe plastic deformation (SPD) techniques such as Equal–Channel Angular Pressing (ECAP) [5] and High-Pressure Torsion (HPT) [6] have been used to refine the grain structure of metallic materials and give grain sizes in the submicrometer or nanometer range that are smaller than those produced using conventional thermo–mechanical processing techniques. The low ductility of magnesium generally precludes the use of ECAP processing at low temperatures and ECAP processing at high temperatures reduces the effectiveness of the grain refinement. By contrast, processing by HPT is associated with high hydrostatic compressive stresses that prevent the failure of brittle materials. This SPD technique has been used widely to process magnesium and its alloys to produce ultrafine grain sizes and increasing strength [7]. There is now experimental evidence that HPT processing enhances the corrosion resistance of pure magnesium [8,9] and also significantly improves the mechanical properties [10,11]. 

Magnesium matrix composites are also potential candidates for structural orthopedic implants where the second phase may improve the strength and/or improve the biological performance. For instance, bioactive glasses (BG) display significant bioactivity and bone-bonding capacity. Calcium phosphates such as hydroxyapatite (HA) are particularly interesting materials for orthopedic applications because HA can form strong chemical bonds with the bone tissue and it is one of the main components affecting the mechanical strength and providing stiffness to bone [3]. However, bone grafts made exclusively of HA may have only restricted applicability because of their high density (3.1 g/cm^3^) and low fracture toughness (0.7 MPa/m^2^). Accordingly, metal-matrix composites consisting of a biodegradable Mg-alloy matrix together with HA particles as a secondary phase are important materials for orthopedic implants because they provide an opportunity to match the biological performance of HA with the toughness of Mg.

There are many reports on the fabrication of magnesium-based composites with hydroxyapatite using not only commercial purity Mg (CP-Mg) [12,13,14,15,16,17,18], but also Mg alloys such as AZ91 (Mg-9 wt% Al-1 wt% Zn) [19,20]. The usual fabrication methods involve heating, such as sintering [12,16,17] or hot extrusion [15,18,19,21]. The addition of HA within the Mg matrix, combined with mechanical processing, provides both grain refinement and a gain in strength. Conversely, a common difficulty when the HA content is above ~10 wt.% is the development of an inhomogeneous distribution of HA [14,15,19] which compromises the corrosion resistance of the composite. 

The use of HPT also permits a fabrication of metal matrix composites (MMCs) through the consolidation of particles [22,23,24]. This method provides an opportunity for producing MMCs without any heating, but with ultrafine grains in the matrix. It was recently shown that it is possible to produce an Mg-Al_2_O_3_ composite with a good dispersion of the ceramic phase [25] and an Mg-Al composite with good ductility [26]. Thus, the present research was undertaken in order to make use of HPT in fabricating magnesium-based bioactive composites and also to provide an initial characterization of the microstructure, mechanical properties, and corrosion of these materials.

## 2. Materials and Methods 

### 2.1. Preparation of the Composites

**Figure 1:**
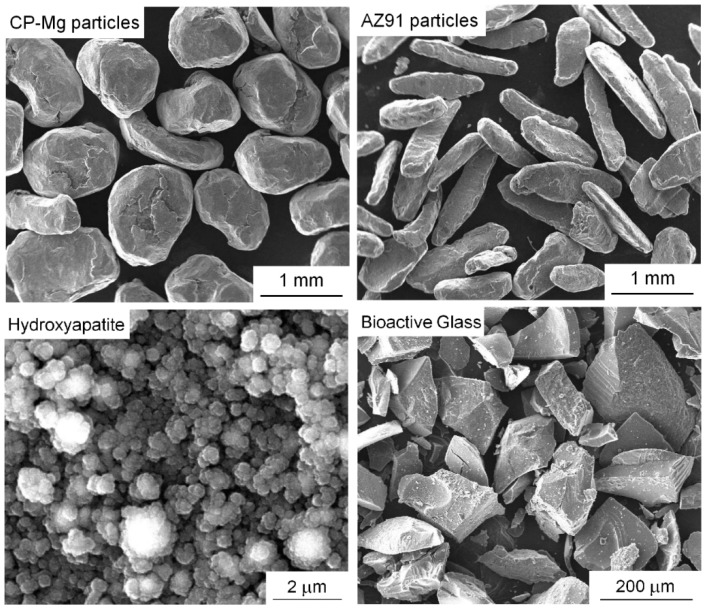
Particles used as starting materials.

The experiments were conducted using particles as starting materials. Commercial purity magnesium and AZ91 magnesium alloy particles were provided by RIMA (Bocaiúva, MG, Brazil). Hydroxyapatite-Ca_5_(OH)(PO4)_3_ (HA) nanopowder (Sigma-Aldrich, Saint Louis, MO, USA) was used as the reinforcement phase. 58S bioactive glass (BG) (60 mol% SiO_2_, 36 mol% CaO, 4 mol% P_2_O_5_) was prepared by the sol-gel process. Tetraethyl orthosilicate (TEOS/ 98%, Sigma-Aldrich), triethyl phosphate (TEP, ≥99.8%, Sigma-Aldrich) and calcium nitrate tetrahydrate (Ca(NO_3_)_2_·4H_2_O, ≥98%, Vetec/Sigma-Aldrich) were used as precursors in these syntheses. The monoliths produced were then hand-milled and air-dried at 120 °C for 5 days before heat-treating in air at 700 °C for 6 h. A detailed description of this procedure and the characterization of the material are given elsewhere [27]. Figure 1 shows secondary electron images (SEI) of the materials used. It is observed that the CP-Mg and the AZ91 particles are significantly coarser than the bioactive materials. The hydroxyapatite nanopowder displays some agglomeration. 

The CP-Mg and AZ91 particles were mixed with 5 wt.% of a bioactive component (HA and BG) creating the blends Mg-5% HA, Mg-5% BG, and AZ91-5% BG, which were initially compacted at room temperature into 10 mm diameter and 1 mm thickness discs using a hydraulic press. The compacted discs were subsequently consolidated at room temperature using a quasi-constrained HPT facility under a nominal pressure of 6 GPa. The anvils used for processing contained a shallow depression of 0.25 mm depth and 10 mm diameter. After processing, the discs exhibited thicknesses in the range 0.6–0.8 mm. The equipment operated at 1 rpm of rotation rate. The Mg-5% BG was processed through 10 turns and the Mg-5% HA and AZ91-5% BG through 50 turns. 

### 2.2. Microstructure Characterization and Mechanical Testing

After HPT processing, the samples were ground with abrasive paper, polished with diamond paste and a colloidal silica solution to obtain a mirror-like finish. Each polished sample was examined by scanning electron microscopy (SEM, Quanta 200 FEI, Hillsboro, USA). Images were collected from both secondary electrons (SE) and backscattered electrons (BSE). The composition of selected areas was estimated using energy dispersive spectroscopy (EDS).

The mechanical properties were evaluated by Vickers hardness testing with a load of 50 gf and a dwell time of 10 s. Additionally, two miniature tensile specimens were cut from symmetrical positions in each Mg-5% HA disc using electro-discharge machining. As in an earlier investigation [28], the gauge sections of the specimens were located at distances of ~2 mm from the disc centers and the specimens had lengths and widths of ~1 mm and thicknesses of ~0.60 mm. Tensile tests were performed at room temperature at a constant cross-head displacement rate using an initial strain rate of 1.0 × 10^−4^ s^−1^. 

### 2.3. Corrosion and Cytotoxicity Tests

The corrosion behavior was assessed by electrochemical impedance spectroscopy (EIS) in Hank’s solution using a potentiostat (Autolab PGSTAT 100N with FRA32M impedance modulus, Metrohm, Herisau, Switzerland) with 3 electrodes (Ag/AgCl as reference, platinum as counter electrode and the composite as the working electrode). The EIS test was performed at frequency range of 1 mHz to 10^4^ Hz and amplitude of 0.01 V using 250 mL of solution. The composition of Hank’s solution is given in Table 1. The discs were ground using #4000 abrasive paper and then immersed in Hank’s solution for at least 600 s before testing. After immersion, the samples were dried and the corroded surface was examined by SEM. In practice, the immersion times varied among samples since the composites with BG displayed faster corrosion than the HA-containing materials. The corrosion rate of the Mg-HA composite was determined by assessing the volume of H_2_ formed during the soaking in 500 mL of Hank’s solution. The electrochemical tests and immersion tests were conducted at room temperature and 37 °C, respectively.

In order to provide an initial evaluation of the incorporation of hydroxyapatite in pure magnesium biocompatibility, the cytotoxicity of the Mg-5% HA composite was estimated using a 3-(4.5-dimethylthiazolyl-2)-2.5-diphenyltetrazolium bromide (MTT) test. Thus, 4 wells were seeded with ~1.0 × 10^5^ fibroblast cells, WI-26 VA4 (ATCC^®®^ CCL-95.1TM) cell lineage, in 500 µL of RPMI 1640 (Sigma-Aldrich) media supplemented with 10% fetal bovine serum (Gibco). The cells were maintained for 24 h at 37 °C and 5% CO_2_ and then washed with 200 µL PBS 1×. Afterwards, 500 µL of media supplemented with 1% fetal bovine serum was added to each well. A sample of the Mg-5% HA composite was placed in one of the wells and kept for 24 h. The cells in the 4 wells were then washed using PBS, a solution of 0.5 ng/mL of MTT was added and they were kept for a further 3 h. The absorbance at 550 nm was measured using a SpectraMax M5E (Molecular Devices, San Jose, CA, USA) spectrophotometer. The cells in the wells not in contact with the composite were considered to be the control group. The absorbance of the cells in contact with the composite was expressed as a fraction of the control group to evaluate the overall viability. 

## 3. Results

### 3.1. Consolidation

**Figure 2:**
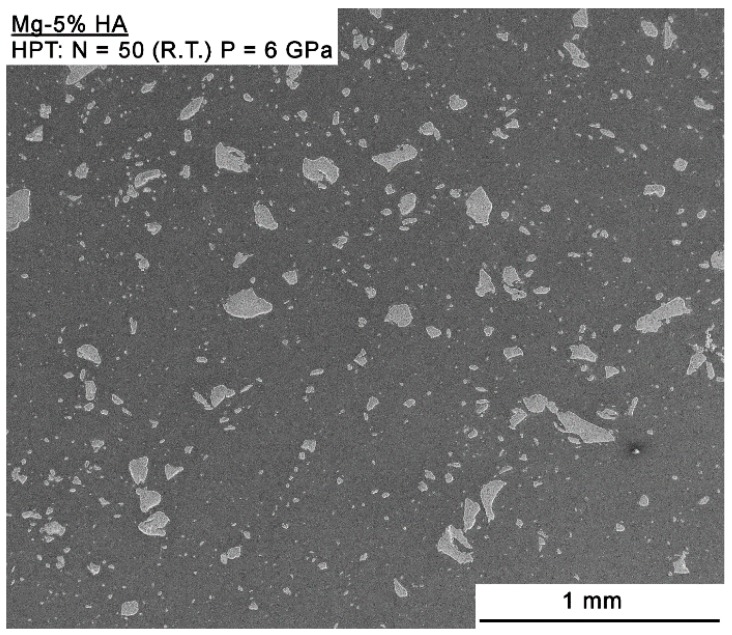
SEM backscattered electron image of the mid-radius area of the Mg-HA composite.

**Figure 3:**
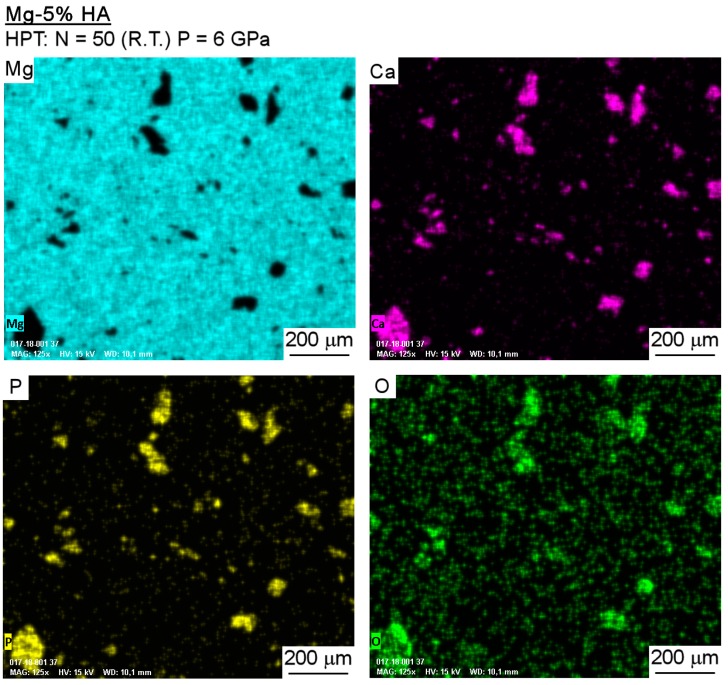
EDS composition maps taken at a selected area of the Mg-HA composite.

Observations using SEM showed good consolidation and the presence of well-dispersed particles in the Mg-HA composite. Figure 2 shows a representative image taken at the mid-radius position. A continuous magnesium matrix and second phase particles having sizes of tens to hundreds of microns is readily observed. Figure 3 depicts EDS compositional maps of the Mg-HA composite where the distribution of Mg, Ca, P, and O reveals a continuous Mg matrix while the second phase is rich in Ca, P, and O.

**Figure 4:**
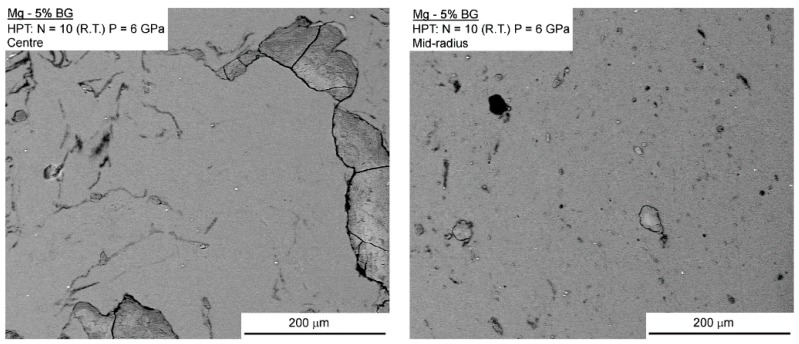
SEM backscattered electron images of areas near the centre (**left**) and at the mid-radius position (**right**) of the Mg-BG composite.

The Mg-BG composite displayed slightly different microstructures near and away from the center of the disc-shaped sample. Thus, Figure 4 shows SEM images demonstrating the presence of coarse second phase particles near the center (left image) and much finer particles at the mid-radius position (right image). A few cracks are visible in the coarse second phase particles near the center suggesting that HPT processing thereby promotes their fragmentation. This observation agrees with the presence of finer particles away from the center where the amount of imposed torsional straining is significantly higher.

**Figure 5:**
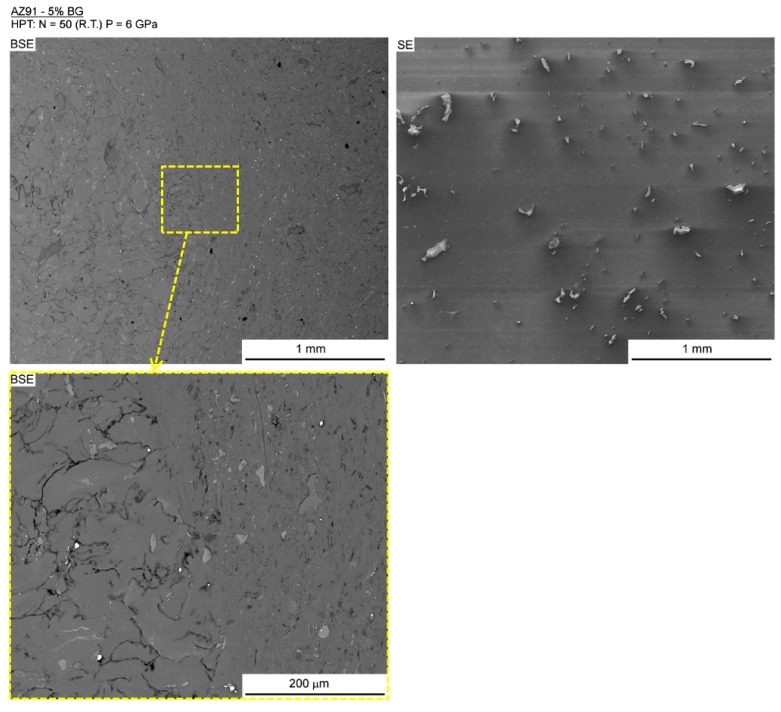
SEM images using backscattered electrons (**left**) and secondary electrons (**right**) of the AZ91-BG composite.

The AZ91-BG composite also exhibited different microstructures at and away from the disc center. Figure 5 shows low magnification images, using backscattered electrons (BSE) and secondary electrons (SE), of a region near the center. The BSE image on the left portrays the presence of coarse volumes with similar sizes to the initial AZ91 particles near the center, suggesting a lack of consolidation in this area. A continuous matrix was observed away from the center, indicating the achievement of full consolidation in this region. A higher magnification image of the boundary between the areas with or without consolidation is also shown. The SE image on the right depicts the distribution of the hard BG particles. As already noted for the Mg-BG composite, finer BG particles were observed away from the center of the AZ91-BG composite.

**Figure 6:**
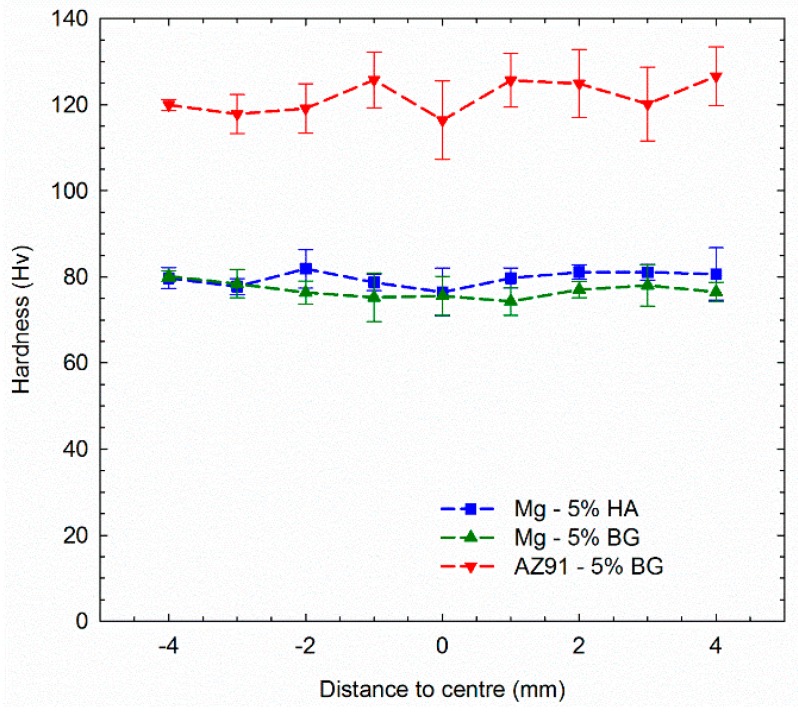
Microhardness distribution along the disc diameter in the different Mg composites.

Figure 6 presents the hardness distributions along randomly selected diameters for the samples obtained in these experiments. The CP-Mg-based composites display a homogeneous distribution of microhardness within the interval of ~75–80 Hv while the AZ91-based composite is harder with values in the range of ~115–125 Hv. These latter values are similar to those recorded in a bulk AZ91 alloy subjected to 5 HPT turns [29] and this shows that the second phase was not effective in increasing the strength of the alloy. Nevertheless, there are some reports of lower hardness values for bulk pure magnesium processed by HPT as, for example, an average hardness of 45 Hv in as-cast magnesium processed from 1/8 to 16 HPT turns [30] and a hardness lower than 40 Hv for pure Mg processed to 10 turns [31]. Machining chips of CP-Mg consolidated using HPT displayed a hardness of ~48 Hv after 5 turns [25], but by contrast, a Mg-Al_2_O_3_ composite consolidated by HPT exhibited a higher hardness (~65 Hv) than the bulk material near the edge of the sample after only five turns [25]. These various results show that the second phase is effective in increasing the hardness of pure magnesium and the higher values of hardness in the current experiments for the CP-Mg-based composites are therefore attributed to the presence of second phase particles and the large numbers of turns.

### 3.2. Corrosion

**Figure 7:**
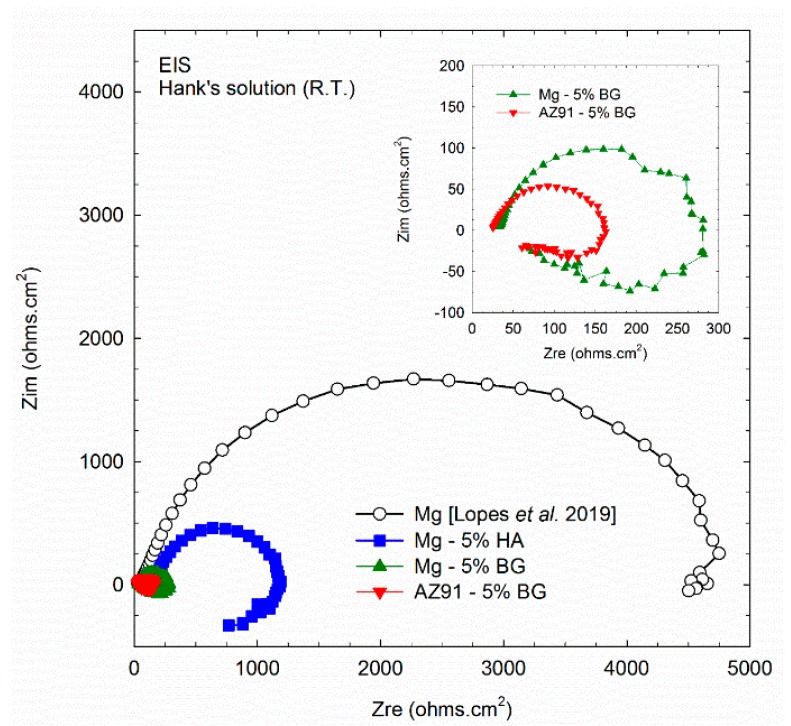
Electrochemical impedance spectroscopy tests in Hank’s solution for the different composites. The value for bulk Mg processed by HPT [32] is also shown for comparison.

The curves obtained by EIS for different composites are presented in Figure 7 where the impedance arc is significantly larger for the Mg-HA composite when compared to the BG-containing composites. For convenience, the arcs for the Mg-BG and AZ91-BG composites are also shown in an insert at the upper right with a reduced scale. A curve obtained during testing of commercial purity Mg (CP-Mg) processed by HPT [32] is also shown in Figure 7 for comparison purposes. Thus, it is apparent that the second phase reduces the corrosion resistance of Mg and this effect is more pronounced when using BG. In fact, the Mg-BG and AZ91-BG composites showed faster corrosion rates and the processed discs were completely corroded in less than 24 h of immersion. It is also worth noting that the AZ91-BG alloy displays an arc smaller than the Mg-BG sample and this is attributed to the presence of precipitates in the AZ91 matrix.

**Figure 8:**
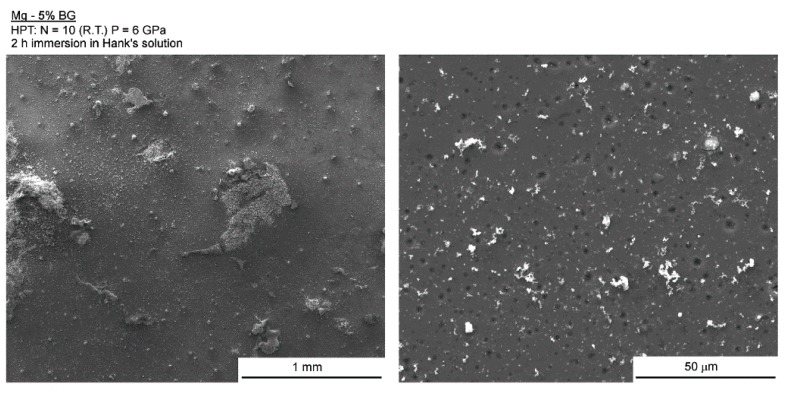
Low (**left**) and high (**right**) magnification images of the surface of the Mg-BG composite after 2 h of immersion in Hank’s solution.

**Figure 9:**
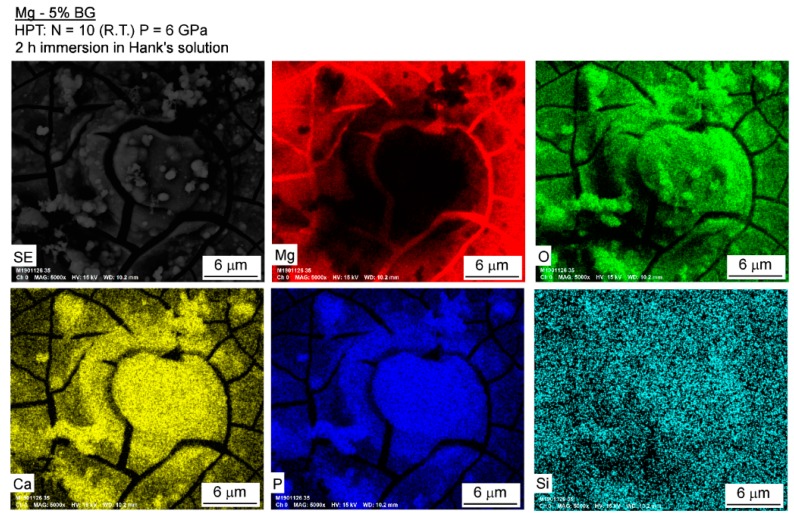
EDS mapping of an area of localized corrosion in the Mg-BG composite after 2 h of immersion in Hank’s solution.

The surfaces of the Mg-BG and AZ91-BG composites were examined using SEM after 2 h of immersion in Hank’s solution. Figure 8 reveals that Mg-BG exhibits large areas of localized corrosion but it is apparent at lower magnification (left) that most of the surface undergoes no significant corrosion. However, at higher magnification (right) it is possible to distinguish many areas of localized corrosion throughout the magnesium matrix. EDS mapping was used to evaluate the composition around one of these areas and Figure 9 displays the distribution of Mg, O, Ca, P, and Si together with the secondary electron image. It is apparent that the volume of the corrosion product in this area is poor in Mg, rich in Ca, P, and O and the distribution of Si is homogeneous but significantly lower than for Ca and P. This suggests the formation of calcium phosphate in the corrosion product.

**Figure 10:**
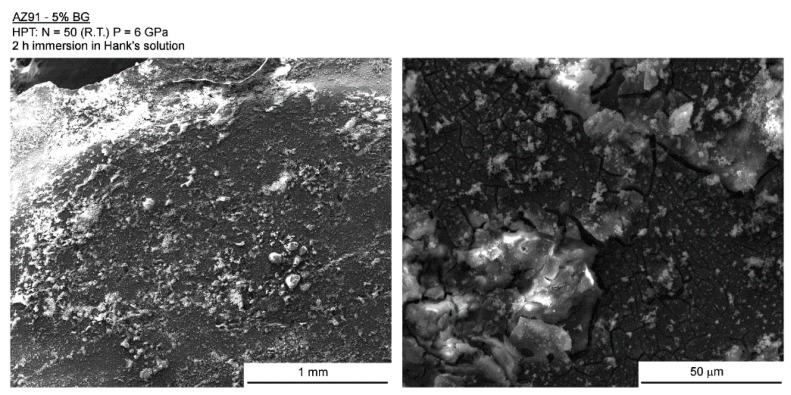
Low (**left**) and high (**right**) magnification images of the surface of the AZ91-BG composite after 2 h of immersion in Hank’s solution.

The AZ91-BG composite showed signs of more severe corrosion throughout the sample surface and Figure 10 portrays the appearance of the surface after 2 h of immersion in Hank’s solution. For this composite, the corrosion product is thicker than observed in the Mg-BG composite and the whole surface of the sample is corroded. 

**Figure 11:**
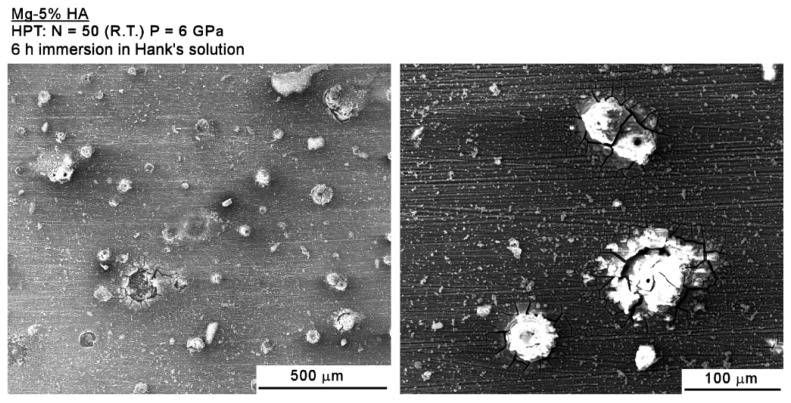
Low (**left**) and high (**right**) magnification images of the surface of the Mg-HA composite after 6 h of immersion in Hank’s solution.

Figure 11 depicts the surface of the Mg-HA composite after 6 h of immersion in Hank’s solution where it is apparent that some areas undergo localized corrosion. While the immersion period was longer in the Mg-HA composite compared to the Mg-BG composites, it is readily apparent that the process of corrosion is slower in the former and the areas of localized corrosion do not effectively propagate.

### 3.3. Mg-Hydroxyapatite Composite

**Figure 12:**
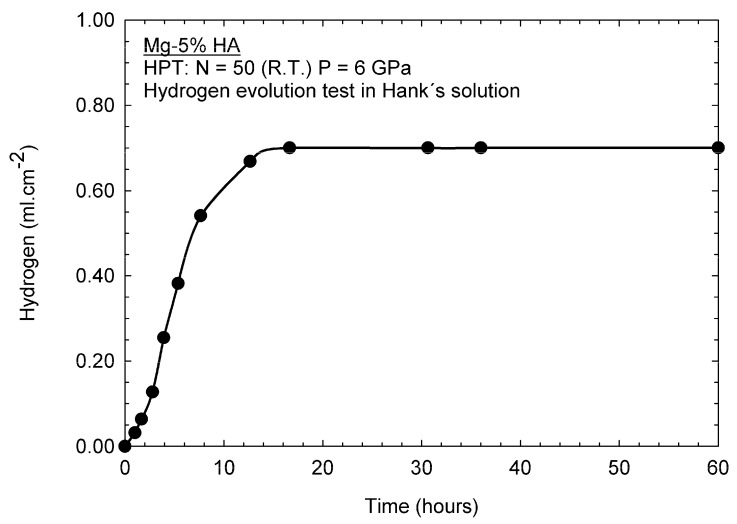
Hydrogen evolution as a function of time for the Mg-HA composite immersed in Hank’s solution.

The Mg-BG and the AZ91-BG samples failed to withstand 24 h of immersion in Hank’s solution at 37 °C. Nevertheless, the Mg-HA composite displayed a decreasing rate of corrosion with immersion time and further tests were therefore conducted with this material. Figure 12 shows the hydrogen evolution rate and thus a fast corrosion rate was observed in the initial testing stage. However, the corrosion rate decreased and ultimately became undetectable after about 15 h of immersion. This suggests the formation of a protective layer of a corrosion product.

**Figure 13:**
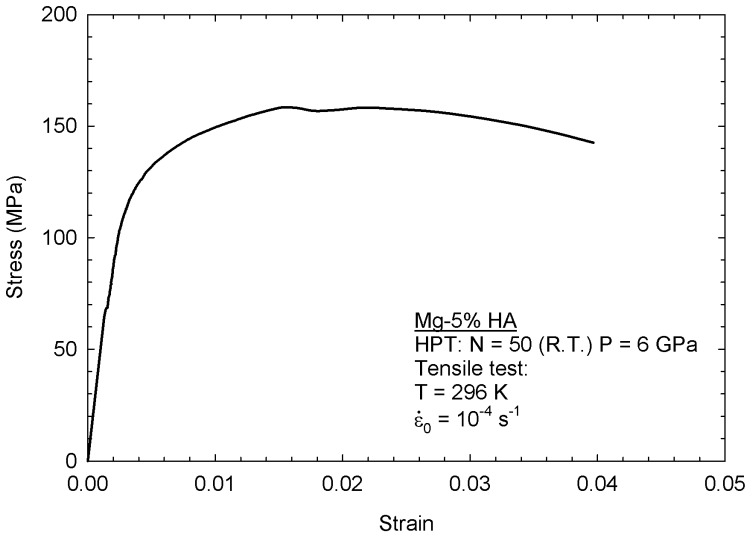
Stress vs. strain curve for the Mg-HA composite.

The effectiveness of the consolidation of Mg particles in the Mg-HA composite was further assessed using miniature tensile testing and Figure 13 shows an engineering stress vs. engineering strain curve obtained for the Mg-5% HA composite processed through 50 turns of HPT and then pulled to failure at room temperature. Thus, the composite displays an ultimate stress over 150 MPa, which is significantly higher than observed in CP-Mg processed by HPT and tested using a similar strain rate [10,11]. The occurrence of this enhanced strength is consistent with the enhanced hardness compared to bulk magnesium and it is attributed to the presence of the second phase particles. However, the ductility is significantly reduced compared to bulk magnesium [10,11,33]. 

The viability of the Mg-HA composite was evaluated using a MTT test. The values of the absorbance measured in the control groups (with no contact with any sample) and the group of cells in contact with the composite for 24 h are presented in Table 2. Thus, the viability of the composite is high, which shows that it is not toxic despite the fast initial corrosion. This is in agreement with an earlier report showing that HPT processing has no effect on the in vitro biocompatibility of pure magnesium and magnesium alloys [32].

## 4. Discussion

### 4.1. The Effectiveness of the Consolidation

The consolidation of metallic particles through HPT processing appears to be straight-forward in principle. Thus, the imposed compressive stress is many times higher than the flow stress of the base metal and this should induce plastic deformation throughout the sample. The torsional strain is sufficiently high to promote an increase in contact area between the particles and this will lead to self-welding. This general effect was reported in the very early experiments of Bridgman [34] where it was claimed that finely divided powder emerges as a homogeneous disc after shearing. In fact, there are many reports of the consolidation of metallic particles by HPT [23,35,36,37,38,39,40,41,42] and initial observations of the processed discs suggest a full consolidation. Earlier reports described good consolidation of pure magnesium machining chips [25,43] after only five turns of HPT. Nevertheless, it was reported that the magnesium alloy AZ91 does not display full consolidation after similar processing [43]. The present results show that, even after 50 turns of HPT under a nominal pressure of 6 GPa, the boundaries between the initial coarse metallic particles remain visible at the center of the AZ91-BG composite and a full consolidation was only observed from the mid-radius to the outer edge of the disc where the imposed torsional strains are significantly higher. The explanation for this lack of welding between particles is attributed to sliding between the particles which is probably aided by the presence of finely-dispersed bioactive glass particles in the present experiments. Additionally, it is known that strain localization may take place during the HPT processing of magnesium alloys [44,45] and this may lead to significantly lower deformation in large volumes of the disc, which will effectively compromise the welding of the particles. 

Despite the lack of full consolidation in the AZ91-based composite, pure magnesium-based composites display a homogeneous metallic matrix throughout the discs which suggests full consolidation. The effectiveness of consolidation was further assessed in the Mg-HA composite by tensile testing. Thus, areas with a lack of bonding should act as internal cracks that will then compromise the tensile resistance of the composite and yet the ultimate stress of the composite is higher than observed in bulk pure magnesium [10,11], which also supports the assumption of a full consolidation. It is worth noting that hardness and compression testing are not as effective as tensile testing in evaluating the soundness of the metallic matrix since internal defects propagate under tensile stresses, but these defects can effectively withstand compressive loading. While there are reports of the fabrication of magnesium-hydroxyapatite composites [12,15,16,17,18,19], tensile testing was seldom conducted and the ultimate stress observed in the present experiments is comparable to that observed in composites produced through hot-extrusion [14,15]. It is concluded, therefore, that HPT processing is effective in producing sound magnesium-based composites without the need to heat the composite components. This approach therefore provides the opportunity to use temperature-sensitive materials to produce magnesium-based composites with a drug delivery ability.

### 4.2. Performance in Hank’s Solution

The EIS tests revealed small impedance arcs for the BG composites which is consistent with the fast corrosion observed in these materials. However, the Mg-HA composite displayed an impedance arc that was larger than observed in conventional AZ31 and ZK60 magnesium alloys [32] and this shows that the Mg-HA composite exhibits a considerable corrosion resistance. The hydrogen evolution test showed that the initially high corrosion rate gradually decreases until it reaches a very low value. While this test is not effective for evaluating low corrosion rates, it shows clearly that there is a trend of decreasing degradation rate and this is supported by the observation that a thin disc (~0.5 mm in thickness) withstood 60 h of immersion in Hank’s solution without developing a hole or any pronounced corrosion. It seems important in future experiments to further investigate the corrosion behavior of this composite. 

While the Mg-BG and AZ91-BG composites displayed fast corrosion, there is evidence for the formation of calcium phosphate on the surface of the AZ91-BG composite. Thus, bioactive glass promotes the nucleation and growth of calcium phosphates in physiological media and this enhances the integration with bone. This bioactivity of BG is also observed in the magnesium-based composite, but further research will be needed in order to enhance the corrosion resistance of this composite and to more fully establish the potential for producing bioactive and biodegradable composites. The poor corrosion resistance displayed by BG-containing composites may be related to the large particle size of BG which was above 200 µm. It appears that the BG exhibited breakage during the HPT processing with no filling of the voids and cracks by the metallic matrix (Figure 4 and Figure 5). It was already reported that these defects can display a negative influence on the corrosion resistance of magnesium in physiological media [46]. Thus, in practice, the particle size of BG could be decreased by, for example, either ball-milling or the use of a different sol-gel route [47].

## 5. Summary and Conclusions

1. Hydroxyapatite and bioactive glass particles were mixed with pure magnesium and particles of a magnesium AZ91 alloy and consolidated into a bulk disc at room temperature using HPT. The integrity of the composites was assessed using SEM examination and tensile testing. EIS and observations of the sample surfaces after immersion in Hank’s solution were used to evaluate the corrosion behavior.
2. Sound composites were produced with a continuous pure magnesium matrix and well-dispersed hydroxyapatite or bioactive glass particles. The AZ91 particles failed to consolidate well in the center of the disc.
3. The composites with bioactive glass exhibited small impedance arcs and fast corrosion. Calcium phosphate was observed on the surface of a sample after only 2 h of immersion in Hank’s solution.
4. The composite with hydroxyapatite displayed a high tensile strength of ~160 MPa and a decreasing rate of corrosion.
5. High-pressure torsion is an effective procedure for consolidating magnesium particles with bioactive components at room temperature.

## Figures and Tables

**Table 1:** Composition of Hank’s solution.

Chemical Compost	Concentration (g·L^−1^)
NaCl	8
KCl	0.4
MgSO_4_·7H_2_O	0.06
MgCl_2_·6H_2_O	0.1
CaCl_2_	0.14
Na_2_HPO_4_·2H_2_O	0.06
KH_2_PO_4_	0.06
Glucose	0.1
NaHCO_3_	0.35

**Table 2:** Citotoxicity of the Mg-5% HA composite evaluated by MTT test.

	Absorbance	Average	Viability
Control Group	2.8065	2.817	100%
	2.9065		
	2.7375		
Mg-5% HA	2.781	2.781	99%
